# The Effects of Menopause Hormone Therapy on Lipid Profile in Postmenopausal Women: A Systematic Review and Meta-Analysis

**DOI:** 10.3389/fphar.2022.850815

**Published:** 2022-04-12

**Authors:** Guangning Nie, Xiaofei Yang, Yangyang Wang, Wanshi Liang, Xuewen Li, Qiyuan Luo, Hongyan Yang, Jian Liu, Jiajing Wang, Qinghua Guo, Qi Yu, Xuefang Liang

**Affiliations:** ^1^ Department of Gynecology, The Second Affiliated Hospital of Guangzhou University of Chinese Medicine, Guangzhou, China; ^2^ The Second Clinical Medical College of Guangzhou University of Chinese Medicine, Guangzhou, China; ^3^ Department of Standardization of Traditional Chinese Medicine, The Second Affiliated Hospital of Guangzhou University of Chinese Medicine, Guangzhou, China; ^4^ State Key Laboratory of Dampness Syndrome of Chinese Medicine, The Second Affiliated Hospital of Guangzhou University of Chinese Medicine, Guangzhou, China; ^5^ Department of Cardiovascular Medicine, Guangdong Provincial Hospital of Chinese Medicine, Guangzhou, China; ^6^ Health Science Center, Shenzhen University, Shenzhen, China; ^7^ Department of Gynecology, Peking Union Medical College Hospital, Beijing, China

**Keywords:** menopause hormone therapy, lipid profile, meta-analysis, postmenopausal women, system review

## Abstract

**Importance:** The incidence of dyslipidemia increases after menopause. Menopause hormone therapy (MHT) is recommended for menopause related disease. However, it is benefit for lipid profiles is inconclusive.

**Objective:** To conduct a systematic review and meta-analysis of randomized controlled trials to evaluate the effects of MHT on lipid profile in postmenopausal women.

**Evidence Review:** Related articles were searched on PubMed/Medline, EMBASE, Web of Science, and Cochrane Library databases from inception to December 2020. Data extraction and quality evaluation were performed independently by two reviewers. The methodological quality was assessed using the “Cochrane Risk of Bias checklist”.

**Results:** Seventy-three eligible studies were selected. The results showed that MHT significantly decreased the levels of TC (WMD: −0.43, 95% CI: −0.53 to −0.33), LDL-C (WMD: −0.47, 95% CI: −0.55 to −0.40) and LP (a) (WMD: −49.46, 95% CI: −64.27 to −34.64) compared with placebo or no treatment. Oral MHT led to a significantly higher TG compared with transdermal MHT (WMD: 0.12, 95% CI: 0.04–0.21). The benefits of low dose MHT on TG was also concluded when comparing with conventional-dose estrogen (WMD: −0.18, 95% CI: −0.32 to −0.03). The results also showed that conventional MHT significantly decreased LDL-C (WMD: −0.35, 95% CI: −0.50 to −0.19), but increase TG (WMD: 0.42, 95%CI: 0.18–0.65) compared with tibolone. When comparing with the different MHT regimens, estrogen (E) + progesterone (P) regimen significantly increased TC (WMD: 0.15, 95% CI: 0.09 to 0.20), LDL-C (WMD: 0.12, 95% CI: 0.07–0.17) and Lp(a) (WMD: 44.58, 95% CI:28.09–61.06) compared with estrogen alone.

**Conclusion and Relevance:** MHT plays a positive role in lipid profile in postmenopausal women, meanwhile for women with hypertriglyceridemia, low doses or transdermal MHT or tibolone would be a safer choice. Moreover, E + P regimen might blunt the benefit of estrogen on the lipid profile.

**Clinical Trial Registration**: [https://www.crd.york.ac.uk/prospero/display_record.php?ID=CRD42018092924], identifier [No. CRD42018092924].

## Introduction

Several studies have shown that menopause transition is associated with an unfavorable effect on lipid profile, accompanying with an increase in the levels of total cholesterol (TC), low-density lipoprotein cholesterol (LDL-C), triglycerides (TG), and lipoprotein (a) [LP (a)], and sometimes with a decrease in the level of high-density lipoprotein cholesterol (HDL-C) (Anagnostis et al., 2015; Anagnostis et al., 2016). It is well-known that an unfavorable lipid profile plays a crucial role in the development and progression of cardiovascular disease (CVD) (McQueen et al., 2008; Lee et al., 2017), which is the leading cause of morbidity and mortality in postmenopausal women (Tandon et al., 2010).

Menopause signifies the permanent cessation of menstruation, resulting from loss of ovarian follicular activity and deficiency of estrogen. As postmenopausal women have significantly higher levels of LDL-C and TC than premenopausal women (Ambikairajah et al., 2019), estrogen has been found to play a protective role by regulating lipid metabolism. In this frame, estrogen-based menopause hormone therapy (MHT) could influence lipid profile in postmenopausal women. It has been reported that MHT is the most effective treatment for menopause-related symptoms caused by the loss of estrogen (Baber et al., 2016). Besides, MHT has been shown to have a favorable risk–benefit ratio for women without dyslipidemia who underwent treatment at the age under 60 years old or within 10 years after menopause onset (2019 Surveillance of Menopause, 2019). A meta-analysis conducted in 2001 concluded that MHT could decrease the levels of TC and LDL-C, and increase HDL-C level (Godsland, 2001). A review performed in 2017 showed that MHT significantly decreased LP (a) concentration (Anagnostis et al., 2017). Some studies have shown that MHT negatively influences TG level (Mercuro et al., 2003; Nii et al., 2016). However, a study conducted in 2016 indicated that TG level was lower in MHT group than that in non-MHT group (Ki et al., 2016). Pu et al. pointed out that hormone therapy with 17*β*-estradiol provided more benefits for decreasing TG level, while conjugated equine estrogen (CEE) showed a better effect on reducing the levels of both HDL-C and LDL-C (Pu et al., 2017). To date, long-term effects of MHT or different routes of administration of estrogen on the lipid profile were scarcely reported. In addition, it has been shown that both dosage and type of progestogen are of great importance for the lipoprotein fractions (Odmark et al., 2004). The Women’s Health Initiative (WHI) study demonstrated that CEEs with medroxyprogesterone acetate (MPA) had an increased risk of developing coronary heart disease (CHD) by 18%, while the CEE was not associated with an increased risk of CHD, raising a question concerning the safety of progestogen (Manson et al., 2013; Manson et al., 2017). But few meta-analyses have concentrated on the effects of progestogen on lipid profile. Given these limitations, an updated meta-analysis is precious to indicate the effects of MHT on the lipid profile. The present study aimed to systematically review and analyze data from randomized controlled trials (RCTs)to find out the effects of MHT concerning factors, including duration of therapy, route of administration, dosage, and types of regimens [estrogen-alone (E-alone) or estrogen plus progestogen (E + P)], on lipid profile in menopausal women.

## Methods

This review was performed according to the Preferred Reporting Items for Systematic Reviews and Meta-analyses (PRISMA) statement checklist (Moher et al., 2009), and that was registered on PROSPERO (Registration No. CRD42018092924).

### Study Selection

PubMed/Medline, EMBASE, Web of Science, and Cochrane Library databases were comprehensively and systematically searched from inception to 31 December 2020, for studies published in English. The main search items were as follows: (“Menopause Hormone Therapy” OR “hormone therapy” OR “estrogen therapy” OR “estradiol therapy”) AND [“TC” OR “TG” OR “LDL” OR “HDL” OR “LP (a)” OR “lipid” AND (“postmenopausal women” OR “menopausal women” OR “menopause” OR “peri-menopausal women”). This review was performed according to the Preferred Reporting Items for Systematic Reviews and Meta-analyses (PRISMA) statement checklist (Moher et al., 2009), and that was registered on PROSPERO (Registration No. CRD42018092924). Two authors screened and evaluated all the abstracts and potentially eligible articles, any discrepancies between reviewers in the study selection were resolved via consultation with a third reviewer.

Articles that meet the following requirements were included: 1) original RCTs that were published in English; 2) administration of MTH for postmenopausal concerning factors, such as duration of therapy, route of administration, dosage, and types of regimens (E-alone or estrogen E + P); 3) inclusion of placebo, no treatment or non-MHT as a control group. For different regimens, regarding the effects of different types of estrogen on lipid profile, the same type of estrogen was required in 2 groups; 4) reporting the levels of TC, TG, LDL, HDL or Lp (a) as the outcome measures for lipid profile, and data were available directly from articles or could be calculated by mathematical formulas. The unit of TC, TG, LDL, and HDL was uniformly converted to mmol/L, and the unit of Lp(a) was converted to mg/L. As tibolone can alleviate menopause symptoms, studies that compared the effects of tibolone with MHT on lipid profile were included, while studies that concentrated on only the effects of tibolone were excluded from this review.

### Data Extraction and Quality Assessment

Data extraction of the studies included: 1) basic data of retrieved articles (title, the first author’s full name, year of publication, journal, etc.); 2)study design; 3) participants’ demographic characteristics (age, number of cases, etc.); 4)inclusion and exclusion criteria particularly for each article; 5) MHT-based data (name, dose, route of administration, the duration of treatment and type of regimen); 6) data related to control group (name, dose, route of administration, duration, type of regimen, etc.); 7) Serum lipid profiles. The data that provided baseline values and percentage changes after treatment only, which was unable to be converted into averages and standard deviations would be excluded. If raw data is needed, the corresponding author would be contacted to get more details. The Cochrane Risk of Bias check list (Higgins et al., 2011) was used to evaluate the risk of bias of randomized clinical trials.

### Statistical Analysis

Data analyzed was performed with the Cochrane Collaboration Review Manager (version 5.2) software, each outcome was expressed as mean ± standard deviation (SD). Heterogeneity among studies was estimated by *I* ^2^ statistic. If *I* ^2^ ≥ 50%, the random-effects model was used to perform the analysis; Otherwise, the fixed-effects model was utilized. We used the methods recommended in the Cochrane Handbook for Systematic Reviews of Interventions (Ver. 6.2) to resolve the post-treatment data in some trials (Higgins et al., 2021). Millimoles per liter (mmol/L) will be used to measure TC, TG, LDL, and HDL while milligrams per liter (mg/L) were used to measure Lp(a).

## Results

**FIGURE 1:**
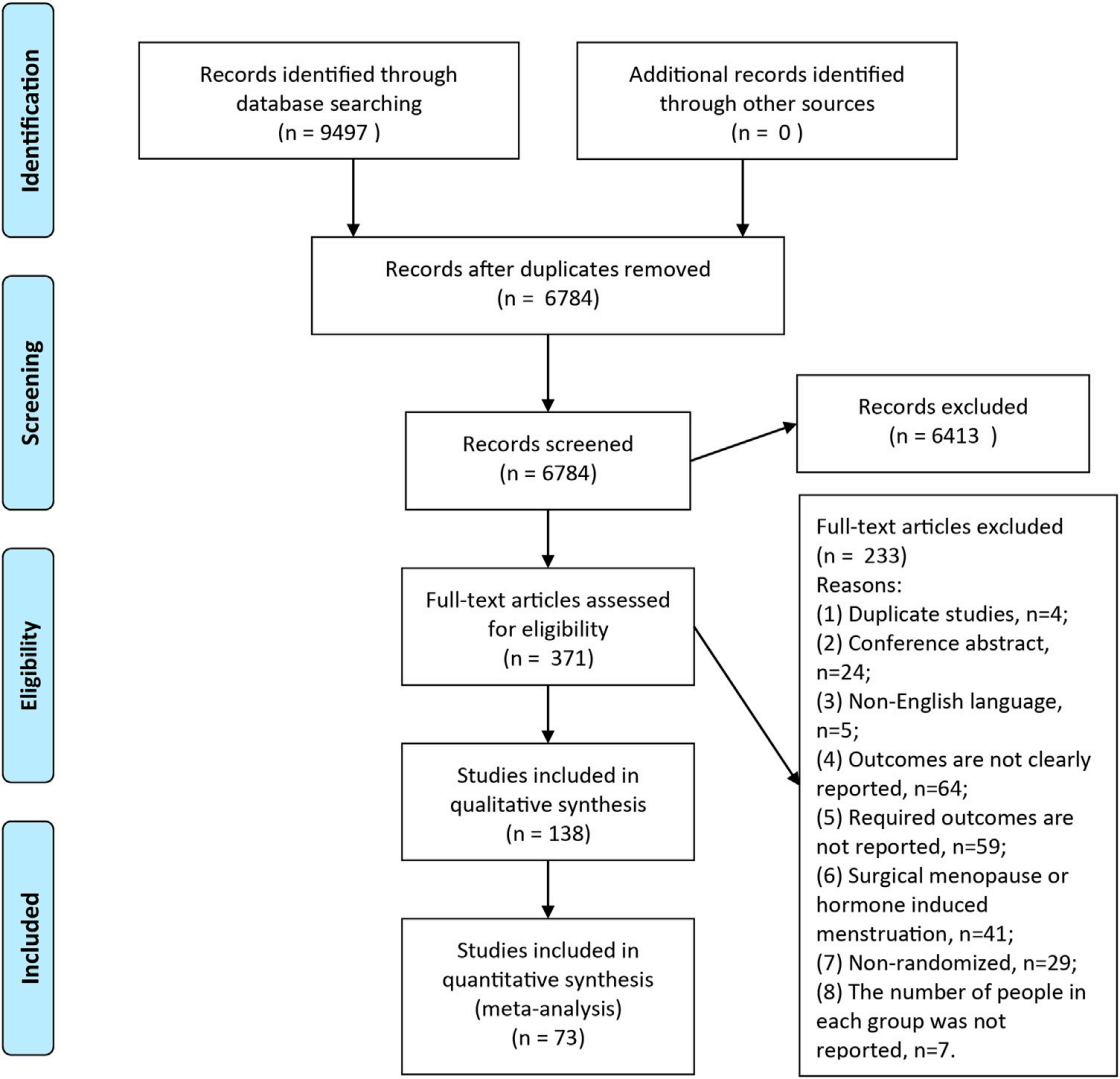
Flow Diagram. A total of 6,784 articles were retrieved, and 73 articles were included in the current meta-analysis.

**FIGURE 2:**
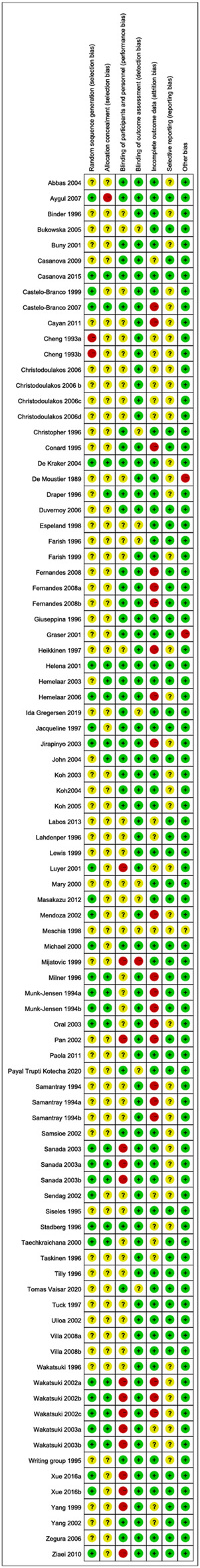
Summary of risk in bias.

**FIGURE 3:**
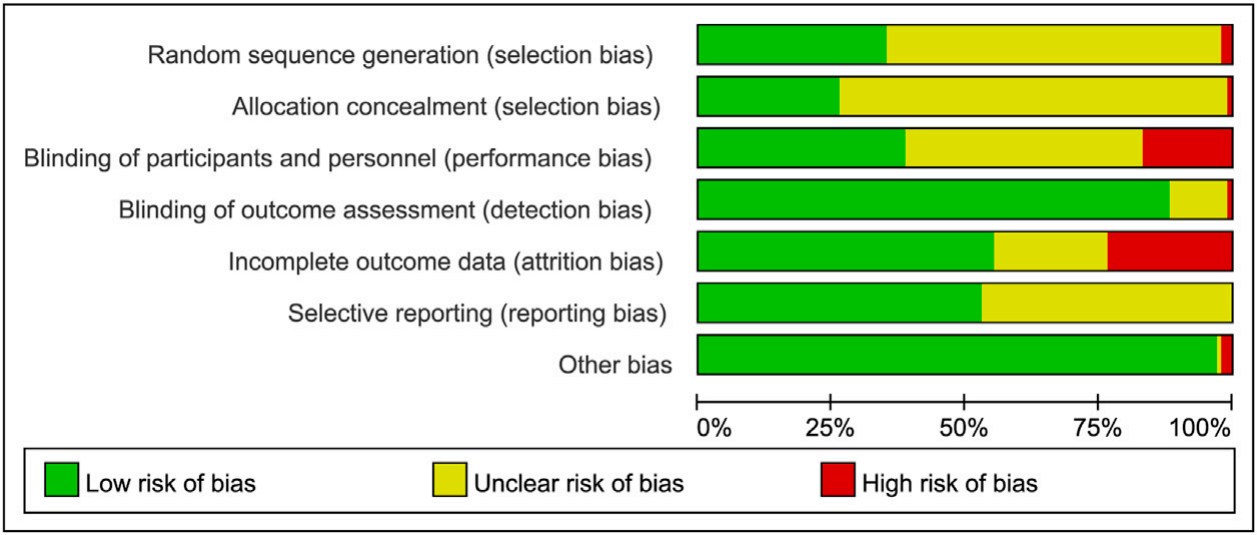
Risk of bias graph.

A total of 9,497 records were searched through database, after removal of duplicates, 6,784 articles were screened full-text and finally 73 articles were included in this meta-analysis (Figure 1). Clinical characteristics of included-articles were described in Table 1. The details for risk of bias are available in Figure 2 and Figure 3.

**TABLE 1:** **Baseline characteristics and clinical outcomes of menopausal women with menopause hormone therapy**.

ID	Author and Year	Control			Treatment			Duration of study (month)	Evaluated Outcomes
		Intervention	*n*	Age (year, Mean ± SD)	Intervention	n	Age (year, Mean ± SD)		
1	Abbas et al. (2004)	Placebo	29	56 ± 2	0.625 mg/day CEE (Oral)	29	56 ± 2	3	HDL-C
		Placebo	29	56 ± 2	Estradiol 100 mcg/day (Transdermal)	29	56 ± 2		
2	Demirol et al. (2007)	Placebo	27	47 ± 0.6	0.625 mg/d CET(Oral)	28	48 ± 0.6	6	Lp(a)
		Placebo	27	47 ± 0.6	2.5 mg/d Tibolone(Oral)	28	46 ± 3	6	Lp(a)
3	Binder et al. (1996)	Placebo	17	67 ± 4	0.625 mg/day CEE + 5 mg MPA(Oral)	15	66 ± 3	11	TC TG HDL LDL
4	Bukowska et al. (2005)	Placebo	16	52.2 ± 3.9	17 beta-estradiol (Transdermal) at increasing-decreasing doses (25, 50, 75, and 50 ug/d) + oralprogesterone 50 to 100 mg	24	52.4 ± 4.8	3	TG HDL Lp(a)
		Placebo	16	52.2 ± 3.9	estradiol valerate 1mg + estriol 2 mg + levonorgestrel 0.25 mg	21	52.3 ± 3.3	3	
5	Bunyavejchevin and Limpaphayom. (2001)	Placebo	26	53.7 ± 4.6	2 mg/day 17 beta-estradiol + 1 mg NETA(Oral)	27	53.4 ± 5.2	12	TC TG HDL LDL
6	Casanova et al. (2009)	3 mg/day 17β-E2 (intranasal route) +200 mg micronized P (vaginal route)	21	51.2 ± 2.7	1 mg/day E2 + 2 mg/daydrospirenone (Oral)	22	51.2 ± 2.7	2	TC TG HDL LDL
7	Casanova et al. (2015a, 2015b)	1.5 mg/day 17β-estradiol gel (percutaneous route or nasal route) +200 mg micronized progesterone (vaginal)	51	51 ± 3	1 mg/day E2 + 2 mg/day drospirenone (Oral)	50	51 ± 3	3	TC TG HDL LDL
8	Castelo-Branco (1999)	Placebo	35	49.9 ± 3.3	0.625 mg/day CEE + 2.5 mg/day medroxyprogesterone/day(Oral)	35	49.0 ± 3.4	24	TC TG HDL LDL
		Placebo	35	49.9 ± 3.3	2.5 mg/day tibolone	35	52.1 ± 3.8		
9	Castelo-Branco (2007)	350 ug/day 17β-estradiol +50 ug/day norethisterone (Intranasal sprays)	94	55 ± 6	2 mg/day 17β-estradiol + 1 mg/dayNETA(Oral)	80	55 ± 6	12	TC TG HDL LDL
		350 ug/day 17β-estradiol +175 ug/day norethisterone (Intranasal sprays)	80	56 ± 5	2 mg/day 17β-estradiol + 1 mg/dayNETA(Oral)	80	55 ± 6		
		350 ug/day 17β-estradiol +550 ug/day norethisterone (Intranasal sprays)	79	56 ± 6	2 mg/day 17β-estradiol + 1 mg/dayNETA(Oral)	80	55 ± 6		
10	Cayan et al. (2011)	No treatment	27	52.3 ± 4.79	0.625 mg/day CEE + 5 mg/day MPA(Oral)	26	50.5 ± 3.4	1	TC HDL LDL TG
		No treatment	27	52.3 ± 4.79	2.5 mg/day tibolone	32	51.5 ± 4.1		
11	Cheng et al. (1993)	Placebo	50	55.9 ± 5.0	2 mg/2 week nylestriol(Oral)	136	54.4 ± 5.7	36	TC TG HDL LDL
		Placebo	50	55.9 ± 5.0	1 mg/2 week nylestriol(Oral)	97	54.8 ± 5.2		
12	Christodoulakos et al. (2006)	No HRT	76	56.3 ± 6.8	0.625 mg/day CEE + 5 mg/day MPA(Oral)	110	53.7 ± 4.2	6	TC TG HDL LDL
		No HRT	76	56.3 ± 6.8	2 mg/day of 17β-estradiol + 1 mg/day NETA(Oral)	76	54.8 ± 4.4		
		No HRT	76	56.3 ± 6.8	1 mg/day of 17β-estradiol + 0.5 mg/day NETA(Oral)	103	56.1 ± 5.1		
		No HRT	76	56.3 ± 6.8	tibolone 2.5 mg	154	55.1 ± 4.3		
13	Haines et al. (1996)	Placebo	45	43.4 ± 5.4	2 mg/d estradiol (Oral)	46	43.8 ± 4.5	6	TC TG HDL LDL Lp(a)
14	Conard et al. (1995)	Placebo	19	51.1 ± 0.9	1 mg/day E2 + 2.5 mg nomegestrolacetate(Oral)	19	52.8 ± 1.0	3	TC TG HDL LDL Lp(a)
		Placebo	19	51.1 ± 0.9	1.5 mg/day E2 + 3.75 mg nomegestrolacetate(Oral)	19	51.5 ± 0.9		
15	de Kraker et al. (2004)	1 mg/day micronised 17β-oestradiol +5 mg dydrogesterone(Oral)	180	54.9 ± 5.1	0.625 mg/day conjugated equine oestrogens +5 mg medroxyprogesterone acetate (Oral)	182	55.1 ± 5.1	12	TC TG LDL
16	Faguer de Moustier et al. (1989)	1.5–3 mg/day of E2 (Percutaneous)	16	/	2 mg/day micronized E2(Oral)	16	/	2	TC TG HDL LDL
17	Draper et al. (1996)	Placebo	64	53.6 ± 3.4	0.625 mg/day CEE(Oral)	64	53.2 ± 3.3	2	TC LDL HDL
18	Duvernoy et al. (2002)	Placebo	9	62 ± 11	10 ug/day ethinyl estradiol + 1 mg/day norethindrone acetate(Oral)	9	62 ± 11	3	TC TG HDL LDL
19	Espeland et al. (1998)	Placebo	72	55.8 ± 4.2	0.625 mg/day CEE(Oral)	74	55.8 ± 4.2	36	Lp(a)
		Placebo	72	55.8 ± 4.2	0.625 mg/day CEE(Oral) + 2.5 mg MPA(Oral)	74	55.8 ± 4.2		
		Placebo	72	55.8 ± 4.2	0.625 mg/day CEE(Oral) + 10 mg MPA (days 1–12, Oral)	73	55.8 ± 4.2		
		Placebo	72	55.8 ± 4.2	0.625 mg/day CEE(Oral) + 200 mg micronized progesterone (Oral ,days 1–12)	73	55.8 ± 4.2		
20	Farish et al. (1999)	2.5 mg/day tibolone	43	53 ± 7	0.625 mg/day CEE + 0.15 mg norgestrel(Oral)	40	52 ± 8	18	TC TG HDL LDL Lp(a)
21	Farish et al. (1996)	oral oestradiol (2 mg/ day)	36	46 + 7	oral oestradiol (2 mg/day) + norethisterone (1 mg/day)	31	45 ± 6	12	TC TG HDL LDL Lp(a)
22	Fernandes et al. (2008)	Placebo	24	52.5 ± 4.8	2 mg/day micronized estradiol(Oral)	25	51.6 ± 3.4	6	TC TG HDL LDL
		Placebo	24	52.5 ± 4.8	2 mg/day micronized estradiol and 1 mg/day norethisterone(Oral)	28	52.1 ± 3.7		
23	Perrone et al. (1996)	No treatment	14	51.8 ± 4.3	0.625 mg/day CEE(Oral) + 10 mg MPA (days 1–12, Oral)	14	51.0 ± 4.1	6	TC TG HDL LDL
		No treatment	14	51.8 ± 4.3	50 µg estradiol (transdermal) + 10 mg MPA (days 1–112, Oral)	14	52.7 ± 3.5		
24	Graser et al. (2001)	Placebo	40	55 ± 5	2 mg/day estradiol valerate + 3 mg/day dienogest(Oral)	43	55 ± 6	6	TC TG HDL LDL
25	Heikkinen et al. (1997)	Placebo	95	52.5 ± 0.22	2 mg/dayEstradiol valerate + 1 mg cyproterone acetate(Oral)	65	52.9 ± 0.29	36	TC TG HDL LDL
26	Teede et al. (2001)	Placebo	30	60 ± 1	2 mg/day oestradiol anhydrous (oral) + 1 mg/day norethisterone acetate (oral)	29	62 ± 2	24	TC TG HDL LDL Lp(a)
27	Hemelaar et al. (2003)	Placebo	49	55.0 ± 4.7	50 µg 17β-estradiol (transdermal)	33	55.5 ± 4.8	17	TC TG HDL LDL Lp(a)
		Placebo	49	55.0 ± 4.7	1 mg 17β-estradiol (oral)	37	54.4 ± 4.3		
		Placebo	49	55.0 ± 4.7	1 mg 17β-estradiol (oral) + 25 µg gestodene	33	53.4 ± 4.2		
28	Hemelaar et al. (2006)	175 ug/day 17β-estradiol +275 ug/dayNET (Intranasal spray)	116	56.8 ± 5.6	1 mg/day 17β-estradiol + 0.5 mg/day NETA(Oral)	117	54.9 ± 4.5	24	TC TG LDL Lp(a) HDL
29	Gregersen et al. (2019)	Placebo	69	55.0 ± 5.2	2 mg/day estradiol and 1 mg/day NETA(Oral)	71	55.5 ± 6.8	24	TC TG HDL LDL Lp(a)
30	Conard et al. (1997)	Placebo	16	54 ± 5	2 mg/day micronized E2(Oral)	20	52 ± 4	6	TC TG HDL LDL Lp(a)
31	Jirapinyo et al. (2003)	Placebo	60	54.6 ± 4.4	2 mg/day E2 + 1 mg/dayNETA(Oral)	60	54.0 ± 4.3	12	TC TG HDL LDL
32	Stevenson et al. (2004)	Placebo	27	56.3 ± 1.2	0.05 mg/dayoestradiol (transdermal) + 0.125 mg/day norethisterone acetatepatches	28	59.8 ± 0.8	6	TC HDL LDL
33	Koh et al. (2003)	Placebo	26	60 ± 1	0.625 mg/day CEE + 100 mg /day MP	53	59 ± 1	2	TC TG HDL LDL
		Placebo	26	60 ± 1	2.5 mg/day tibolone	53	59 ± 1		
34	Koh et al. (2004)	100 mgMP/day + 0.3 mg/day CEE(Oral)	57	57 ± 1	100 mg MP/day + 0.625 mg/day CEE(Oral)	57	57 ± 1	2	TC TG HDL LDL
35	Koh et al. (2005)	2.5 mg/day tibolone	41	59.4 + 1.0	100 mgMP/day + 0.3 mg/day CEE(Oral)	41	59.4 + 1.0	2	TC TG HDL LDL
36	Labos et al. (2013)	No treatment	36	50.56 ± 5.798	1 mg/day 17β-estradiol + 0.5 mg/day norethisterone acetate(Oral)	26	51.50 ± 4.123	12	TC TG HDL LDL
37	Lahdenperä et al. (1996)	0.05 mg/day 17-beta-estradiol (Transdermal) +10 mg medroxyprogesterone acetate(Oral)	41	52.6 ± 2.0	2 mg/day 17-beta-estradiol and 1 mg/day norethisterone acetate (Oral)	36	52.3 ± 2.0	12	TC TG HDL LDL
38	Lewis-Barned et al. (1999)	Placebo	16	52 ± 3	2 mg/day 17β-estradiol + 1 mg norethisterone (Oral)	16	52 ± 3	—	—
39	Luyer et al. (2001)	Placebo	12	65.3 ± 8.0	0.625 mg /day CEE(Oral)	13	68.5 ± 7.0	3	TC TG HDL LDL
40	Davidson et al. (2000)	No treatment	83	58.7 ± 5.2	2 mg/day Oestradiol + 1 mg norethisterine (Day 17–28)	23	58.2 ± 6.7	—	—
		No treatment	83	58.7 ± 5.2	2 mg/day Oestradiol + 700 ug norethisterine	22	58.2 ± 6.7		
		No treatment	83	58.7 ± 5.2	50 ug/day Oestradiol + 170 ug norethisterine (Day 14–28)	33	58.2 ± 6.7		
		No treatment	83	58.7 ± 5.2	50 ug/day Oestradiol + 100 mg testoserone	34	51.7 ± 3.8		
41	Terauchi et al. (2012)	Placebo	67	53.0 ± 4.1	0.5 mg/day mE2	72	52.9 ± 3.6	2	TC TG HDL LDL
		Placebo	67	53.0 ± 4.1	1.0 mg/day mE2	71	52.8 ± 4.6		
42	Mendoza et al. (2002)	2.5 mg/day tibolone	55	50.7 ± 4.2	50 ug/day 17β- oestradiol + 0.25 mg NETA(Transdermal)	55	49.6 ± 3.6	12	TC TG HDL LDL
		2.5 mg/day tibolone	55	50.7 ± 4.2	50 g/day 17-oestradiol (transdermal) + 200 mg progesterone 2/w (oral)	55	50.6 ± 4.2		
43	Meschia et al. (1998)	Placebo	41	53 ± 4.2	50 µg 17β-estradiol (transdermal) + 10 mg MPA (days 1–12)	60	52 ± 4.3	3	TC TG HDL LDL Lp(a)
		Placebo	41	53 ± 4.2	0.625 mg/day CEE(Oral) + 10 mg MPA (days 1–12)	60	51 ± 4.4		
44	Seed et al. (2000)	Placebo	66	57.1 ± 6.8	1 mg/day 17β-estradiol	67	58.6 ± 5.6	6	TC TG HDL LDL Lp(a)
		Placebo	66	57.1 ± 6.8	1 mg/day 17β-estradiol + 0.25 mg norethisterone acetate	68	58.1 ± 5.8		
		Placebo	66	57.1 ± 6.8	1 mg/day 17β-estradiol + 0. 5 mg norethisterone acetate	63	57.7 ± 6.2		
45	Mijatovic et al. (1999)	No treatment	13	53.2 ± 3.5	1 mg/day micronized E2 (Oral) + 5 mg/day or 10 mg/day dydrogesterone	14	51.4 ± 4.0	15	Lp(a)
46	Milner et al. (1996)	Placebo	50	55.6 ± 0.61	0.625 mg/day CEE + 0.15 mg norgestrel (Oral)	32	52.4 ± 0.74	24	TC TG HDL LDL Lp(a)
		Placebo	50	55.6 ± 0.61	2.5 mg/day tibolone	31	53.6 ± 0.77		
47	Munk-Jensen et al. (1994)	Placebo	38	/	Combination: 2 mg/day of 17β-estradiol + 1 mg/day NETA (Oral)	37	/	24	TC TG HDL LDL
		Placebo	38	/	Sequential: 2 mg/day of 17β-estradiol + 1 mg NETA (Oral)	38	/		
48	Oral and Ozbaşar (2003)	Placebo	28	65 ± 1.9	0.625 mg/day CEE + 5 mg/day MPA(Oral)	30	64 ± 2.1	18	TC TG HDL LDL
49	Pan et al. (2002)	2.5 mg/day tibolone	17	51.2 ± 4.3	0.625 mg/day CEE (Oral)	23	52.5 ± 3.4	6	TC TG HDL LDL
50	Kotecha et al. (2020)	placebo	34	60.5 (57.1, 65.4)	2 mg/day 17β-estradiol + 1 mg/day norethisterone acetate (Oral)	34	60.7 (57.3, 62.8)	24	TC TG HDL LDL Lp(a)
		placebo	34	60.5 (57.1, 65.4)	2.5 mg tibolone	33	61.0 (57.7, 65.0)	24	TC TG HDL LDL Lp(a)
51	Villa et al. (2011)	placebo	20	51.9 ± 2.4	1 mg/day E2dose (oral) + drospirenone	20	52 ± 3.3	6	TC TG HDL LDL Lp(a)
52	Samantray KV et al. (1994)	placebo	15	48.4 ± 2.6	0 625 mg/day CEE (Oral)	15	47.7 ± 3.1	3	TC TG HDL LDL
		placebo	15	48.4 ± 2.6	0.625 mg/day CEE + 2.5 mg/day MPA (Oral)	15	49.3 ± 2.8		
53	Samsioe et al. (2002)	placebo	40	56.2 ± 4.6	1 mg/day E2 + 0.25 mg/day NETA (Oral)	40	55.6 ± 4.3	12	TC TG HDL LDL Lp(a)
		placebo	40	56.2 ± 4.6	1 mg/day E2 + 0.5 mg/day NETA (Oral)	40	56.7 ± 5.1		
54	Sanada et al. (2003)	No treatment	15	54.8 ± 4.8	0.625 mg/day CEE + 2.5 mg MPA (Oral)	18	55.1 ± 5.2	3	TC TG HDL LDL
		No treatment	15	54.8 ± 4.8	0.3 mg/day CEE + 2.5 mg MPA (Oral)	18	55.3 ± 5.3		
55	Sendag et al. (2002)	0.05 mg/day 17β estradiol +0.25 mg norethindrone acetate (Transdermal)	42	47.36 ± 3.8	0.625 CEE mg/day + 10 MPA mg (Oral)	42	47.57 ± 3.9	6	TC TG HDL LDL
56	Siseles et al. (1995)	2.5 mg/day tibolone	13	/	5 mg MPA + 0.625 mg/day CE(Oral)	11	/	6	TC TG HDL LDL
								6	TC TG HDL LDL
57	Stadberg et al. (1996)	1 mg E2/day + 0.25 mg/day NETA(Oral)	19	58.5	2 mg E2/day + 1 mg/day NETA(Oral)	21	58.5	12	TC TG HDL LDL Lp(a)
		1 mg E2/day + 0.5 mg/day NETA(Oral)	20	58.5	2 mg E2/day + 1 mg/day NETA(Oral)	21	58.5		
58	Taechakraichana et al. (2000)	30 ug/day ethinyl E2 + 150 ug desogestre(Oral)	40	51.0 ± 0.6	0.625 mg/day CEE + 5 mg medrogestone(Oral)	40	52.3 ± 0.6	12	TC TG HDL LDL
59	Taskinen et al. (1996)	50 μg/day 17β-estradiol(Transdermal) +10 mg MPA	57	52.3 ± 2.3	2 mg/day 17β-estradiol + 1 mg NETA(Oral)	55	52.5 ± 2.5	12	TC TG HDL LDL
60	Tilly-Kiesi et al. (1996)	50 μg/day 17β-estradiol(Transdermal) +10 mg MPA	38	52.6 ± 2.0	2 mg/day 17β-estradiol and 1 mg/day norethisterone acetate(Oral)	37	52.3 ± 2.1	12	TC TG HDL LDL
61	Vaisar et al. (2021)	Placebo	56	50.7 (48,53)	100 ug/day estradiol (Transdermal)	45	51.1 (48,53)	6	TC TG HDL LD
62	Tuck et al. (1997)	Placebo	15	54.5 ± 6.1	0.625 mg/day CEE(Oral)	15	54.5 ± 6.1	6	TC TG HDL LDL
63	Ulloa et al. (2002)	Placebo	11	55.1 ± 1.2	0.625 mg/dayCEE + 5 mg MPA(Oral)	17	53.8 ± 1.0	2	TC TG HDL LDL
64	Villa et al. (2008)	Placebo	16	53.54 + 3.7	1 mg/day E2 + 10 mg MPA(Oral)	16	52.44 + 3.2	3	TC TG HDL LDL
		Placebo	16	53.54 + 3.7	2 mg/day E2 + 10 mg MPA(Oral)	16	54.5 + 4.1		
65	Wakatsuki and Sagara (1996)	0.625 mg/day CEE(Oral)	28	/	0.625 mg/day CEE(Oral) + 2.5 mg MPA(Oral)	21	/	3	TC TG HDL LDL
		0.625 mg/day CEE(Oral)	28	/	0.625 mg/day CEE(Oral) + 5 mg MPA(Oral)	21	/		
66	Wakatsuki et al. (2002)	No treatment	12	53.4 ± 7.3	0.625 mg/day CEE(Oral)	16	52.4 ± 3.3	3	TC TG HDL LDL
		No treatment	12	53.4 ± 7.3	50 μg/day 17β -estradiol(Transdermal)	16	54.7 ± 5.9		
67	Wakatsuki et al. (2003)	No treatment	14	53.4 ± 7.3	0.3125 mg/day CEE(Oral)	17	54.8 ± 6.8	3	TC TG HDL LDL
		No treatment	14	53.4 ± 7.3	0.625 mg/day CEE(Oral)	15	54.8 ± 7.3		
68	Miller et al.,1995	placebo	174	/	0.625 mg/dayCEE(Oral)	175	/	36	TC TG HDL LDL
		placebo	174	/	0.625 mg/dayCEE(Oral) + cyclic 10 mg/day MPA (12 d/month)	174	/		
		placebo	174	/	0.625 mg/dayCEE(Oral) + 2.5 mg/day MPA	174	/		
		placebo	174	/	0.625 mg/dayCEE(Oral) + cyclic 200 mg/day micronized progesterone (12d/month)	178	/		
69	Xue et al. (2016)	0.3 mg/day CEE + 100 mg MP(Oral)	35	53.7 ± 4.2	0.625 mg/day CEE + 100 mg MP(Oral)	37	53.1 ± 3.1	12	TC TG HDL LDL
		0.3 mg/day CEE + 100 mg MP(Oral)	35	53.7 ± 4.2	0.625 mg/day CEE + 10 mg dydrogesterone(Oral)	35	53.4 ± 4.5		
70	Yang et al. (1999)	2.5 mg/day tibolone	20	50.90 ± 3.42	0.625 mg/day CE + 5 mg MPA(Oral)	20	51.80 ± 3.09	6	TC TG HDL LDL
71	Yang et al. (2002)	placebo	18	50.5 ± 2.79	2 mg/day 17β-estradiol + 1 mg/day norethisterone acetate(Oral)	22	51.5 ± 3.70	4	TC TG HDL LDL
72	Zegura et al. (2006)	Placebo	30	55.4 ± 6.4	2 mg/day E2(Oral)	20	49.2 ± 4.0	6	TC TG LDL Lp(a) HDL
		Placebo	30	55.4 ± 6.4	50 μg/day E2(Transdermal)	21	47.8 ± 4.1		
		Placebo	30	55.4 ± 6.4	2 mg/day E2 + 1 mg/day NETA(Oral)	31	55.1 ± 5.3		
73	Ziaei et al. (2010)	Placebo	50	52.52 ± 4.06	0.625 mg/day CEE + 2.5 mg MPA(Oral)	50	51.58 ± 2.82	6	TG HDL
		Placebo	50	52.52 ± 4.06	2.5 mg/day tibolone	50	51.78 ± 3.29		

Abbreviation: CEE, conjugated equine estrogen; MPA, medroxyprogesterone acetate; E2, Estradiol; SD, Standard Deviation

### Comparing the Effects of MHT on Lipid Profile With Placebo or no Treatment

Forty-seven studies (Cheng et al., 1993; Munk-Jensen et al., 1994; Samantray KV et al., 1994; Conard et al., 1995; Miller et al.,1995; Binder et al., 1996; Draper et al., 1996; Haines et al., 1996; Milner et al., 1996; Perrone et al., 1996; Conard et al., 1997; Heikkinen et al., 1997; Tuck et al., 1997; Espeland et al., 1998; Meschia et al., 1998; Lewis-Barned et al., 1999; Mijatovic et al., 1999; Davidson et al., 2000; Seed et al., 2000; Bunyavejchevin and Limpaphayom, 2001; Gräser et al., 2001; Luyer et al., 2001; Teede et al., 2001; Duvernoy et al., 2002; Samsioe et al., 2002; Ulloa et al., 2002; Wakatsuki et al., 2002; Yang et al., 2002; Hemelaar et al., 2003; Jirapinyo et al., 2003; Oral and Ozbaşar, 2003; Sanada et al., 2003; Wakatsuki et al., 2003; Stevenson et al., 2004; Bukowska et al., 2005; Zegura et al., 2006; Demirol et al., 2007; Fernandes et al., 2008; Villa et al., 2008; Ziaei et al., 2010; Cayan et al., 2011; Villa et al., 2011; Terauchi et al., 2012; Labos et al., 2013; Gregersen et al., 2019; Kotecha et al., 2020; Vaisar et al., 2021) compared the effects of MHT therapy and placebo on blood lipids. The duration of MHT was classified into the following periods: < 3 months, 3–5 months, 6–12 months, 13–24 months, and >24 months. For articles that evaluated the effects of MHT on lipid profile at multiple time points, the result in each time point was included as separate data.

**FIGURE 4:**
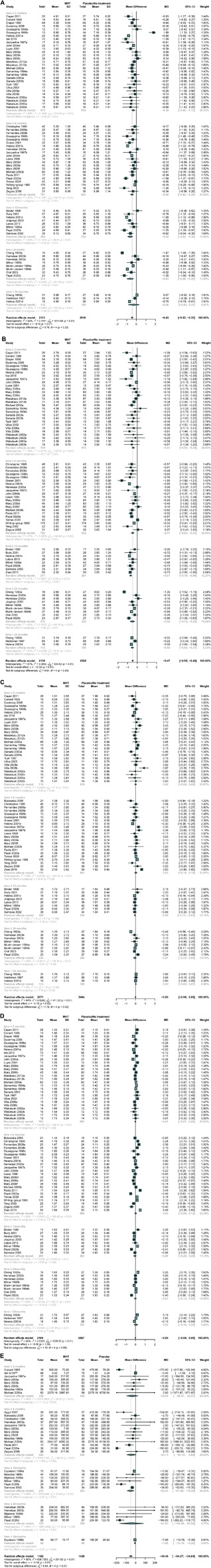
Comparing MHT wih placebo or no treatment. The treatment duration was classified into the following periods in each lipid index: < 3 months, 3–5 months, 6–12 months, 13–24 months, and >24 months. MHT led to a significant reduction in TC concentration, LDL-C concentration and Lp(a) concentration compared with placebo or no treatment. **(A)** TC concentration; **(B)** LDL-C concentration; **(C)** TG concentration; **(D)** HDL-C concentration; **(E)** Lp(a) concentration.

The meta-analysis of data demonstrated that intake MHT could significantly reduce the serum TC (Miller et al., 1995; Binder et al., 1996; Bunyavejchevin and Limpaphayom, 2001; Cayan et al., 2011; Cheng et al., 1993; Conard et al., 1995; Conard et al., 1997; Davidson et al., 2000; Draper et al., 1996; Duvernoy et al., 2002; Fernandes et al., 2008; Gräser et al., 2001; Gregersen et al., 2019; Haines et al., 1996) (WMD: −0.43, 95% CI: −0.53 to −0.33, *I* ^2^ = 93%) (Figure 4A) and LDL (Miller et al., 1995; Binder et al., 1996; Bunyavejchevin and Limpaphayom, 2001; Cayan et al., 2011; Cheng et al., 1993; Conard et al., 1995; Conard et al., 1997; Davidson et al., 2000; Draper et al., 1996; Duvernoy et al., 2002; Fernandes et al., 2008; Gräser et al., 2001; Gregersen et al., 2019; Haines et al., 1996) (WMD: −0.47, 95% CI: −0.55 to −0.40, *I* ^2^ = 87%) throughout almost all treatment duration (Figure 4B). Except the duration between half year to 1 year (WMD: −0.08, 95% CI: −0.13 to −0.03), there was no significant difference in reducing TG (Cheng et al., 1993; Conard et al., 1995; Miller et al., 1995; Binder et al., 1996; Conard et al., 1997; Davidson et al., 2000; Bunyavejchevin and Limpaphayom, 2001; Bukowska et al., 2005; Cayan et al., 2011), (Duvernoy et al., 2002), (Haines et al., 1996; Heikkinen et al., 1997; Gräser et al., 2001; Fernandes et al., 2008; Gregersen et al., 2019) between the two groups (WMD: −0.00, 95% CI: −0.06 to 0.05, *I* ^2^ = 84%) (Figure 4C). While come to Lp(a) (Bukowska et al., 2005; Conard et al., 1995; Conard et al., 1997; Davidson et al., 2000; Demirol et al., 2007; Espeland et al., 1998; Gregersen et al., 2019; Haines et al., 1996; Hemelaar et al., 2003; Kotecha et al., 2020; Meschia et al., 1998; Mijatovic et al., 1999; Milner et al., 1996; Samsioe et al., 2002), the results showed that MHT could remarkably decrease Lp(a) (WMD: −49.46, 95% CI: −64.27 to −34.64, *I* ^2^ = 89%) (Figure 4E). However, the similar trend was only observed in periods of 6–12 months and >24 months. Data from 43 studies suggested an ignorable change in HDL (Miller et al., 1995; Binder et al., 1996; Bukowska et al., 2005; Bunyavejchevin and Limpaphayom, 2001; Cayan et al., 2011; Cheng et al., 1993; Conard et al., 1995; Conard et al., 1997; Davidson et al., 2000; Draper et al., 1996; Duvernoy et al., 2002; Fernandes et al., 2008; Gräser et al., 2001; Gregersen et al., 2019) (WMD: −0.00, 95% CI: −0.05to 0.05, *I* ^2^ = 94%) (Figure 4D).

### Comparing the Effects of Oral MHT With Transdermal MHT

**FIGURE 5:**
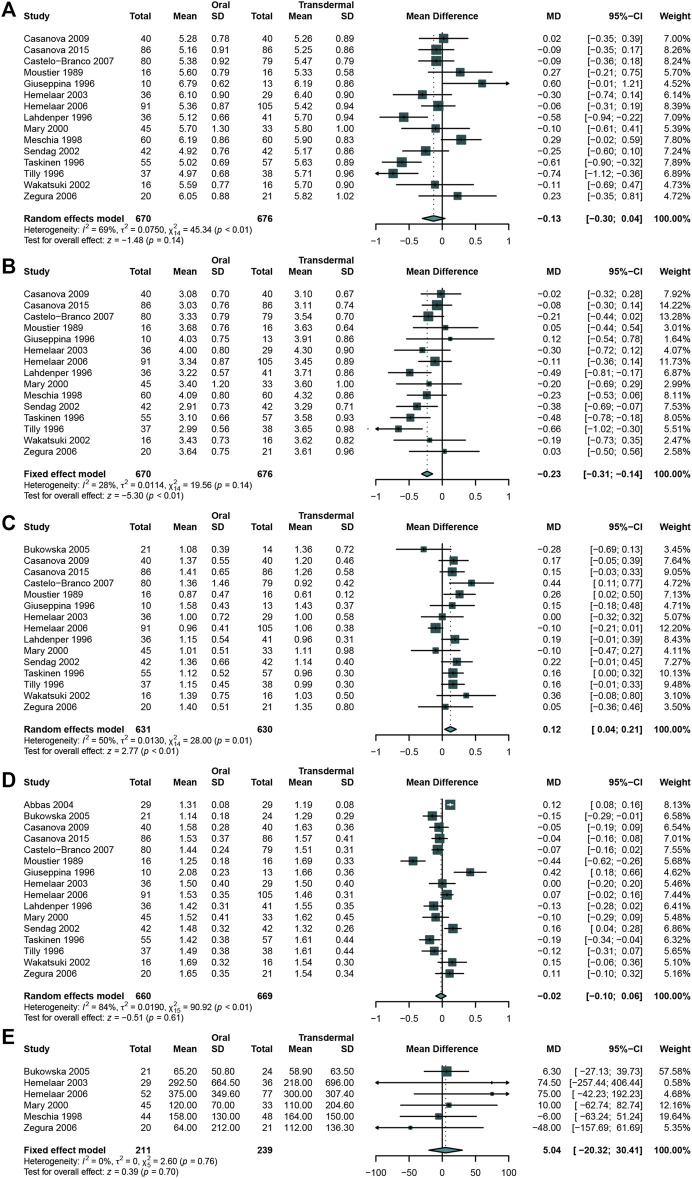
Comparing oral estrogen with transdermal estrogen Oral MHT significantly decreased LDL-C concentration and increased TG concentration compared with that in transdermal MHT group. **(A)** TC concentration; **(B)** LDL-C concentration; **(C)** TG concentration; **(D)** HDL-C concentration; **(E)** Lp(a) concentration.

A total of 16 studies (Hemelaar et al., 2003; Bukowska et al., 2005; Meschia et al., 1998; Perrone et al., 1996; Seed et al., 2000; Zegura et al., 2006; Casanova et al., 2015; Casanova et al., 2009; Castelo-Branco et al., 2007; Faguer de Moustier et al., 1989; Hemelaar et al., 2006; Lahdenperä et al., 1996; Sendag et al., 2002; Taskinen et al., 1996; Tilly-Kiesi et al., 1996; Abbas et al., 2004) that enrolled 670 participants in oral MHT group and 676 in transdermal MHT group were analyzed. When comparing the effects between 2 groups, the result indicated that oral MHT could significantly decreased LDL-C (Hemelaar et al., 2003; Meschia et al., 1998; Perrone et al., 1996; Seed et al., 2000; Casanova et al., 2015; Zegura et al., 2006; Casanova et al., 2009; Castelo-Branco et al., 2007; Faguer de Moustier et al., 1989; Hemelaar et al., 2006; Lahdenperä et al., 1996; Sendag et al., 2002; Taskinen et al., 1996; Tilly-Kiesi et al., 1996) (WMD: 0.23, 95%CI: −0.31 to −0.14, I^2^ = 28%) (Figure 5B) while there was no significant difference in TC (Hemelaar et al., 2003; Meschia et al., 1998; Perrone et al., 1996; Seed et al., 2000; Casanova et al., 2015; Casanova et al., 2009; Zegura et al., 2006; Castelo-Branco et al., 2007; Faguer de Moustier et al., 1989; Hemelaar et al., 2006; Lahdenperä et al., 1996; Sendag et al., 2002; Taskinen et al., 1996; Tilly-Kiesi et al., 1996) (WMD: −0.13, 95% CI: −0.30 to 0.04, *I* ^2^ = 69%) (Figure 5A). However, the result revealed that oral MHT may significantly increase TG (Bukowska et al., 2005; Hemelaar et al., 2003; Perrone et al., 1996; Seed et al., 2000; Casanova et al., 2015; Casanova et al., 2009; Zegura et al., 2006; Castelo-Branco et al., 2007; Faguer de Moustier et al., 1989; Hemelaar et al., 2006; Lahdenperä et al., 1996; Sendag et al., 2002; Taskinen et al., 1996; Tilly-Kiesi et al., 1996) (WMD: 0.12, 95% CI: 0.04 to 0.21, I^2^ = 50%) (Figure 5C), while both HDL (Bukowska et al., 2005; Hemelaar et al., 2003; Perrone et al., 1996; Seed et al., 2000; Casanova et al., 2015; Casanova et al., 2009; Zegura et al., 2006; Castelo-Branco et al., 2007; Faguer de Moustier et al., 1989; Hemelaar et al., 2006; Lahdenperä et al., 1996; Sendag et al., 2002; Taskinen et al., 1996; Abbas et al., 2004) (WMD: -0.02, 95% CI: −0.10 to 0.06, *I* ^2^ = 84%) (Figure 5D) and Lp(a) (Meschia et al., 1998; Seed et al., 2000; Hemelaar et al., 2003; Bukowska et al., 2005; Hemelaar et al., 2006; Zegura et al., 2006) (WMD: 5.04, 95% CI: −20.32 to 30.41, *I* ^2^ = 0%) had no significance (Figure 5E).

### Comparing the Effects of a Low-Dose Estrogen With a Conventional-Dose of Estrogen

The studies were classified according to the dosage of estrogen. A total of 10 studies (Cheng et al., 1993; Stadberg et al., 1996; Taechakraichana et al., 2000; Sanada et al., 2003; Wakatsuki et al., 2003; de Kraker et al., 2004; Koh et al., 2004; Christodoulakos et al., 2006; Villa et al., 2008; Xue et al., 2016)that enrolled 584 participants in low-dose estrogen group and 594 in conventional dose estrogen group were analyzed. 1mg/day or less of Estradiol valerate or 17 *β*-estradiol, 0.3 mg/day or less of conjugated estrogens were defined as low dose estrogen.

**FIGURE 6:**
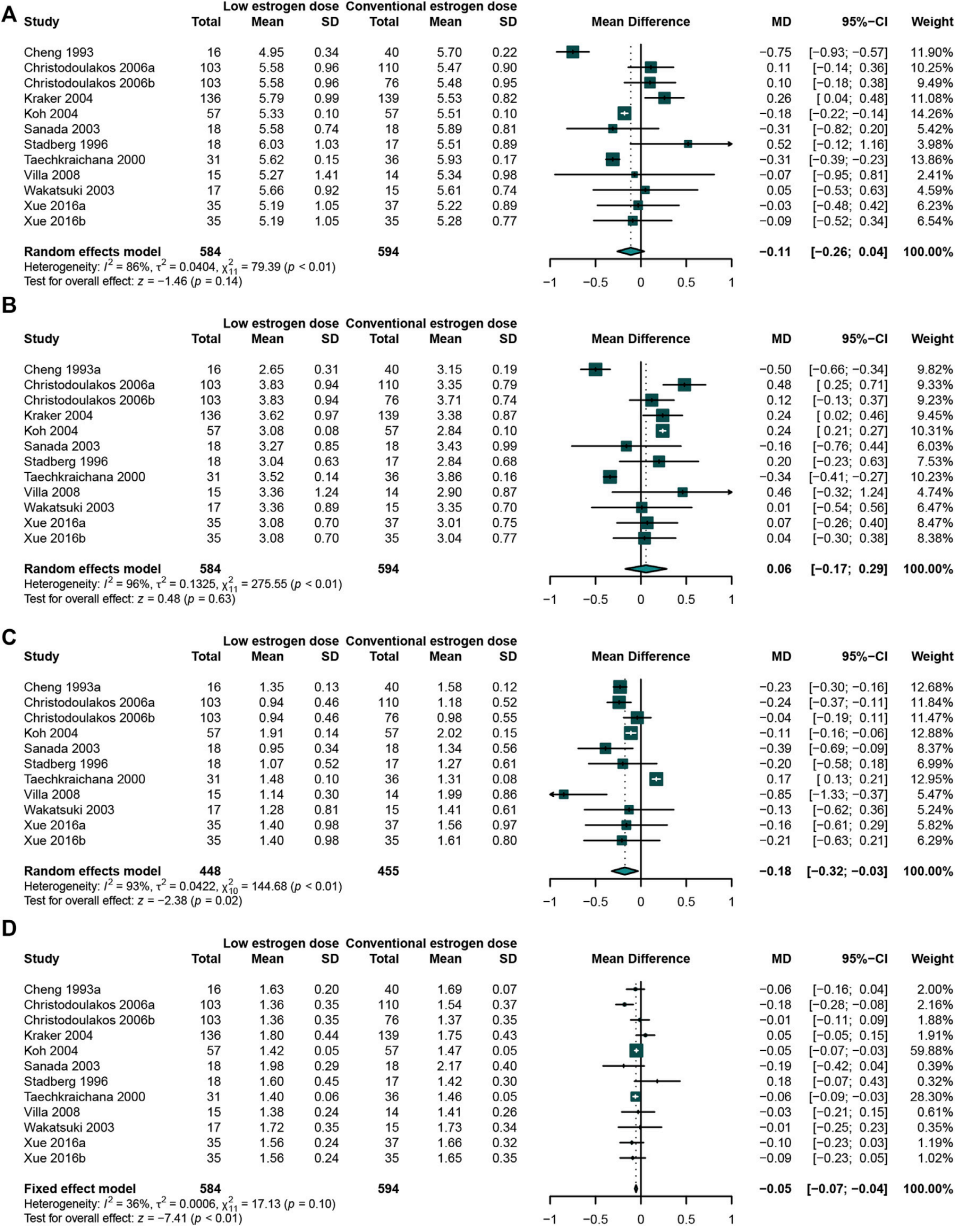
Studies comparing low-dose estrogen with conventional-dose estrogen. A low-dose estrogen led to a significant reduction in TG concentration compared with a conventional-dose estrogen. **(A)** TC concentration; **(B)** LDL-C concentration; **(C)** TG concentration; **(D)** HDL-C concentration; **(E)** Lp(a) concentration.

The meta-analysis result showed that the low-dose estrogen led to a significant reduction in TG (Cheng et al., 1993; Sanada et al., 2003; Villa et al., 2008; Wakatsuki et al., 2003; Christodoulakos et al., 2006; Stadberg et al., 1996; Koh et al., 2004; Taechakraichana et al., 2000; Xue et al., 2016) (WMD: −0.18, 95% CI: −0.32 to −0.03, *I* ^2^ = 93%) (Figure 6C) and HDL-C (Cheng et al., 1993; Sanada et al., 2003; Villa et al., 2008; Wakatsuki et al., 2003; Christodoulakos et al., 2006; de Kraker et al., 2004; Stadberg et al., 1996; Koh et al., 2004; Taechakraichana et al., 2000; Xue et al., 2016) (WMD: −0.05, 95% CI: −0.07 to −0.04, *I* ^2^ = 36%) (Figure 6D) comparing with the conventional-dose estrogen. There was no significant on TC (Cheng et al., 1993; Sanada et al., 2003; Villa et al., 2008; Wakatsuki et al., 2003; Abbas et al., 2004; Christodoulakos et al., 2006; de Kraker et al., 2004; Stadberg et al., 1996; Koh et al., 2004; Taechakraichana et al., 2000) (WMD: −0.11, 95% CI: −0.26 to 0.04, *I* ^2^ = 86%) (Figure 6A) and LDL-C (Cheng et al., 1993; Stadberg et al., 1996; Taechakraichana et al., 2000; Sanada et al., 2003; Wakatsuki et al., 2003; de Kraker et al., 2004; Koh et al., 2004; Christodoulakos et al., 2006; Villa et al., 2008; Xue et al., 2016) (WMD: 0.06, 95% CI: −0.17 to 0.29, I^2^ = 96%) (Figure 6B). Because of only one study evaluated the effects of different doses on Lp(a), meta-analysis was not carried out.

### Comparing the Effects of Conventional MHT With Tibolone

**FIGURE 7:**
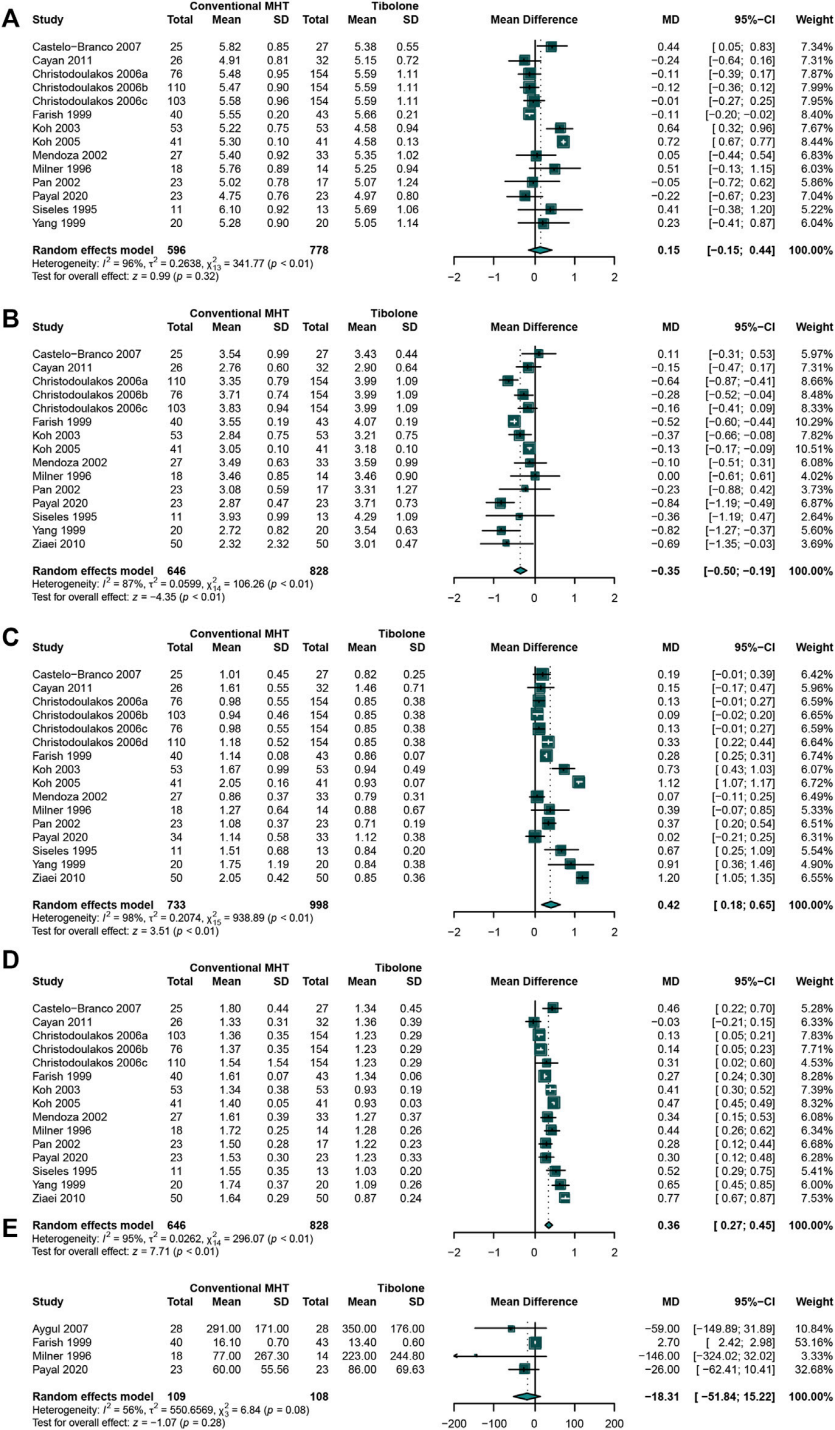
Studies comparing conventional MHT with Tibolone. The conventional MHT could decrease LDL-C concentration, increase TG concentration and HDL-C concentration compared with Tibolone. **(A)** TC concentration; **(B)** LDL-C concentration; **(C)** TG concentration; **(D)** HDL-C concentration; **(E)** Lp(a) concentration.

As tibolone is widely used in mitigating the menopause symptoms, it is necessary to compare the effects of conventional MHT therapy with tibolone on lipids profile. A total of 13 studies (Cayan et al., 2011; Kotecha et al., 2020; Milner et al., 1996; Ziaei et al., 2010; Christodoulakos et al., 2006; Castelo-Branco et al., 1999; Farish et al., 1999; Koh et al., 2003; Koh et al., 2005; Mendoza et al., 2002; Pan et al., 2002; Siseles et al., 1995; Yang et al., 1999)that enrolled 646 participants in conventional MHT group and 828 in tibolone group were analyzed. The outcomes of meta-analysis presented the significantly increasing TG (Cayan et al., 2011; Kotecha et al., 2020; Milner et al., 1996; Ziaei et al., 2010; Christodoulakos et al., 2006; Castelo-Branco et al., 1999; Farish et al., 1999; Koh et al., 2003; Koh et al., 2005; Mendoza et al., 2002; Pan et al., 2002; Siseles et al., 1995; Yang et al., 1999) (WMD:0.42, 95%CI: 0.18 to 0.65, I^2^ = 98%) (Figure 7C) and HDL-C (Cayan et al., 2011; Kotecha et al., 2020; Milner et al., 1996; Ziaei et al., 2010; Christodoulakos et al., 2006; Castelo-Branco et al., 1999; Farish et al., 1999; Koh et al., 2003; Koh et al., 2005; Mendoza et al., 2002; Pan et al., 2002; Siseles et al., 1995; Yang et al., 1999) (WMD: 0.36, 95% CI: 0.27 to 0.45, I^2^ = 95%) (Figure 7D) concentration while significantly decreasing LDL-C (Cayan et al., 2011; Kotecha et al., 2020; Milner et al., 1996; Ziaei et al., 2010; Christodoulakos et al., 2006; Castelo-Branco et al., 1999; Farish et al., 1999; Koh et al., 2003; Koh et al., 2005; Mendoza et al., 2002; Pan et al., 2002; Siseles et al., 1995; Yang et al., 1999) (WMD: −0.35, 95% CI: −0.50 to −0.19, I^2^ = 87%) (Figure 7B) concentration in conventional MHT group. No significant difference was identified in TC (Cayan et al., 2011; Kotecha et al., 2020; Milner et al., 1996; Christodoulakos et al., 2006; Castelo-Branco et al., 1999; Farish et al., 1999; Koh et al., 2003; Koh et al., 2005; Mendoza et al., 2002; Pan et al., 2002; Siseles et al., 1995; Yang et al., 1999) (WMD: 0.15, 95% CI: −0.15 to 0.44, I^2^ = 96%) (Figure 7A) and Lp(a) (Milner et al., 1996; Farish et al., 1999; Demirol et al., 2007; Kotecha et al., 2020) (WMD: −18.31, 95% CI: −51.84 to 15.22, I^2^ = 56%) (Figure 7E) concentration between two groups.

### Comparing the Effects of Estrogen alone (E-Alone) With Estrogen–Progestogen(E + P) Regimen

In total, 8 studies (Samantray KV et al., 1994; Miller et al., 1995; Farish et al., 1996; Wakatsuki and Sagara, 1996; Davidson et al., 2000; Hemelaar et al., 2003; Zegura et al., 2006; Fernandes et al., 2008) that enrolled 836 participants in E-alone group and 818 in E + P group met the criteria of eligibility. The micronized progesterone was used in 2 studies as separate group (Miller et al., 1995; Espeland et al., 1998) and synthetic progestogen was utilized in all these 8 studies.

**FIGURE 8:**
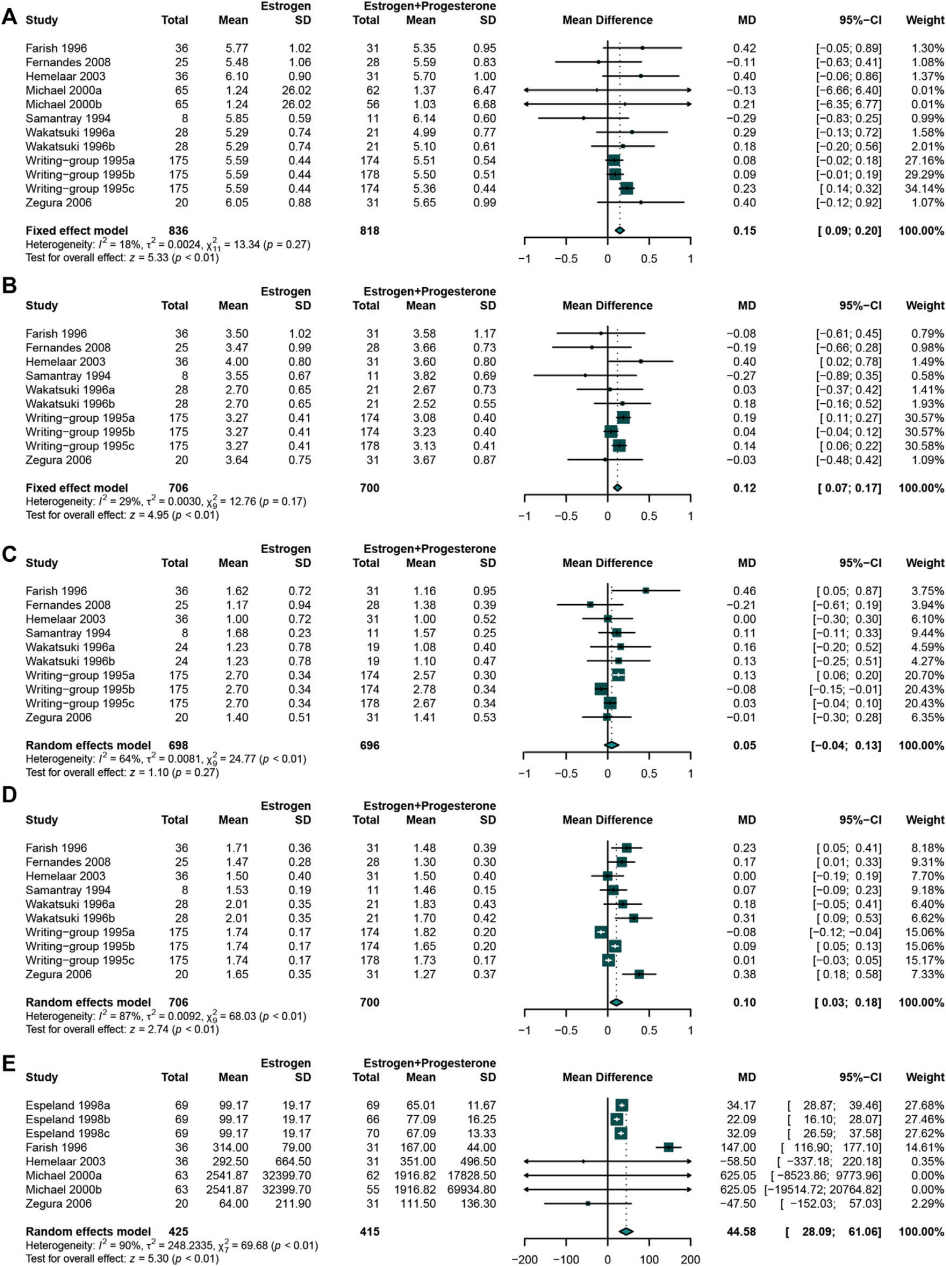
Studies comparing estrogen alone with estrogen plus progestogen regimen. The estrogen plus progestogen regimen could significantly increased TC, LDL-C, HDL-C, and Lp(a) concentration compared with estrogen alone. **(A)** TC concentration; **(B)** LDL-C concentration; **(C)** TG concentration; **(D)** HDL-C concentration; **(E)** Lp(a) concentration.

The results revealed that E + P regimen significantly increased the concentration of TC (Miller et al., 1995; Davidson et al., 2000; Fernandes et al., 2008; Hemelaar et al., 2003; Samantray KV et al., 1994; Zegura et al., 2006; Farish et al., 1996; Wakatsuki and Sagara, 1996) (WMD: 0.15, 95% CI: 0.09 to 0.20, *I* ^2^ = 18%) (Figure 8A), LDL-C (Miller et al., 1995; Fernandes et al., 2008; Hemelaar et al., 2003; Samantray KV et al., 1994; Zegura et al., 2006; Farish et al., 1996; Wakatsuki and Sagara, 1996) (WMD: 0.12, 95% CI: 0.07 to 0.17, *I* ^2^ = 29%) (Figure 8B), HDL-C (Miller et al., 1995; Fernandes et al., 2008; Hemelaar et al., 2003; Samantray KV et al., 1994; Zegura et al., 2006; Farish et al., 1996; Wakatsuki and Sagara, 1996) (WMD: 0.10, 95% CI: 0.03 to 0.18, *I* ^2^ = 87%) (Figure 8D), and Lp(a) (Farish et al., 1996; Espeland et al., 1998; Davidson et al., 2000; Hemelaar et al., 2003; Zegura et al., 2006) (WMD: 44.58, 95% CI:28.09 to 61.06, I^2^ = 90%) (Figure 8E) concentration compared with E-alone. No significant difference was found in TG (Miller et al., 1995; Fernandes et al., 2008; Hemelaar et al., 2003; Samantray KV et al., 1994; Zegura et al., 2006; Farish et al., 1996; Wakatsuki and Sagara, 1996) concentration between these two groups (WMD: 0.05, 95% CI: −0.04 to 0.13, I^2^ = 64%) (Figure 8C).

### Sensitivity Analysis and Publication Bias Assessment

**FIGURE 9:**
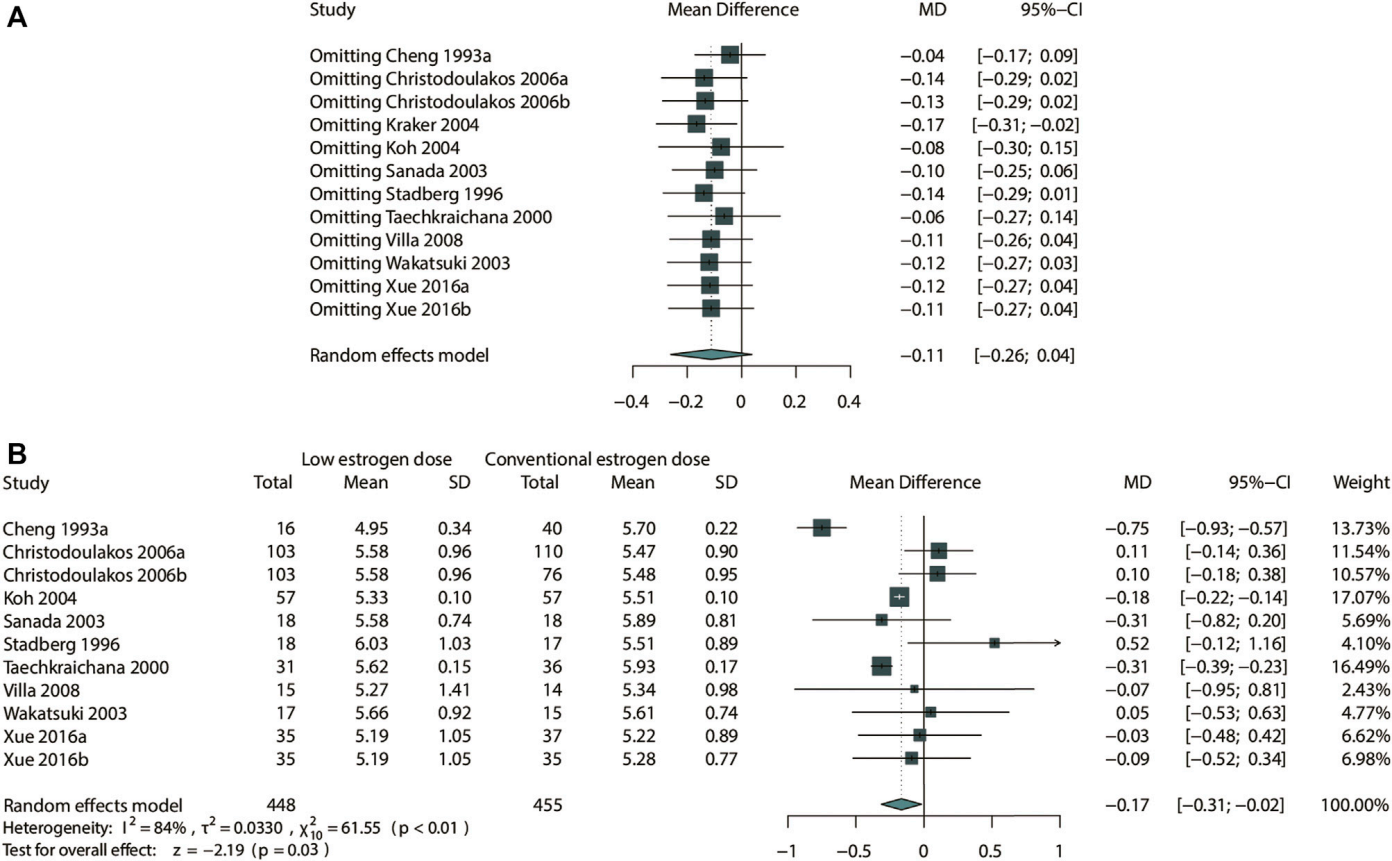
Sensitivity analysis for TC in the subgroup of low-dose estrogen. Sensitivity analysis suggested that while omitting the study Kraker 2004, low-dose estrogen could decrease TC significantly (MD: −0.17 mmol/L, 95% CI: −0.31 to −0.02 mmol/L). **(A)** Sensitivity analysis; **(B)** forrest plot after omitted study Kraker 2004.

**FIGURE 10:**
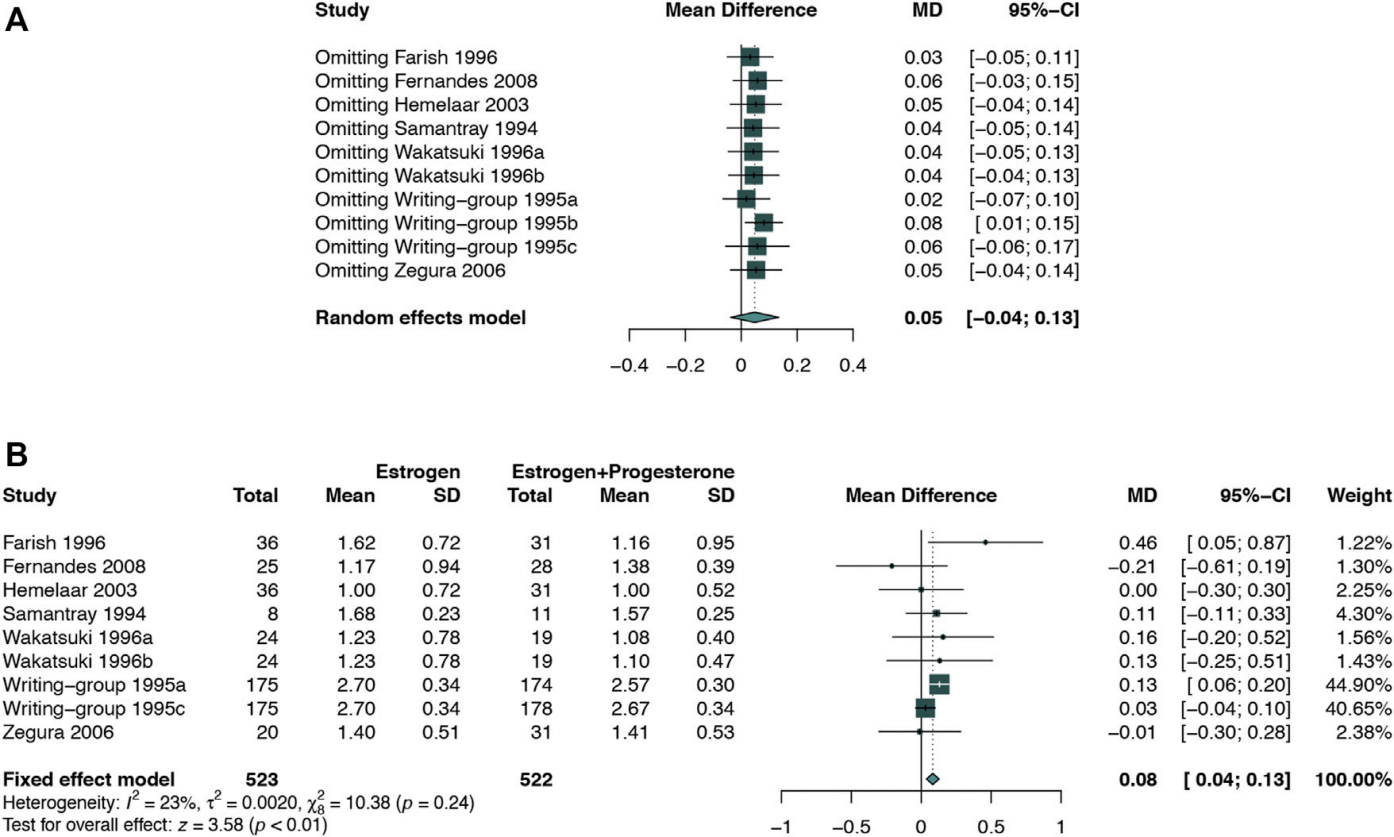
Sensitivity analysis for TG in the subgroup of estrogen alone vs. E + P regimen. Sensitivity analysis suggested that while omitting one group of the study Writing−group 1995b, E + P group cause a significantly higher TG (WMD: 0.08 mmol/L, 95% CI: 0.01–0.15 mmol/L) than Estrogen alone. **(A)** Sensitivity analysis; **(B)** forest plot after omitted study Writing−group 1995b.

**FIGURE 11:**
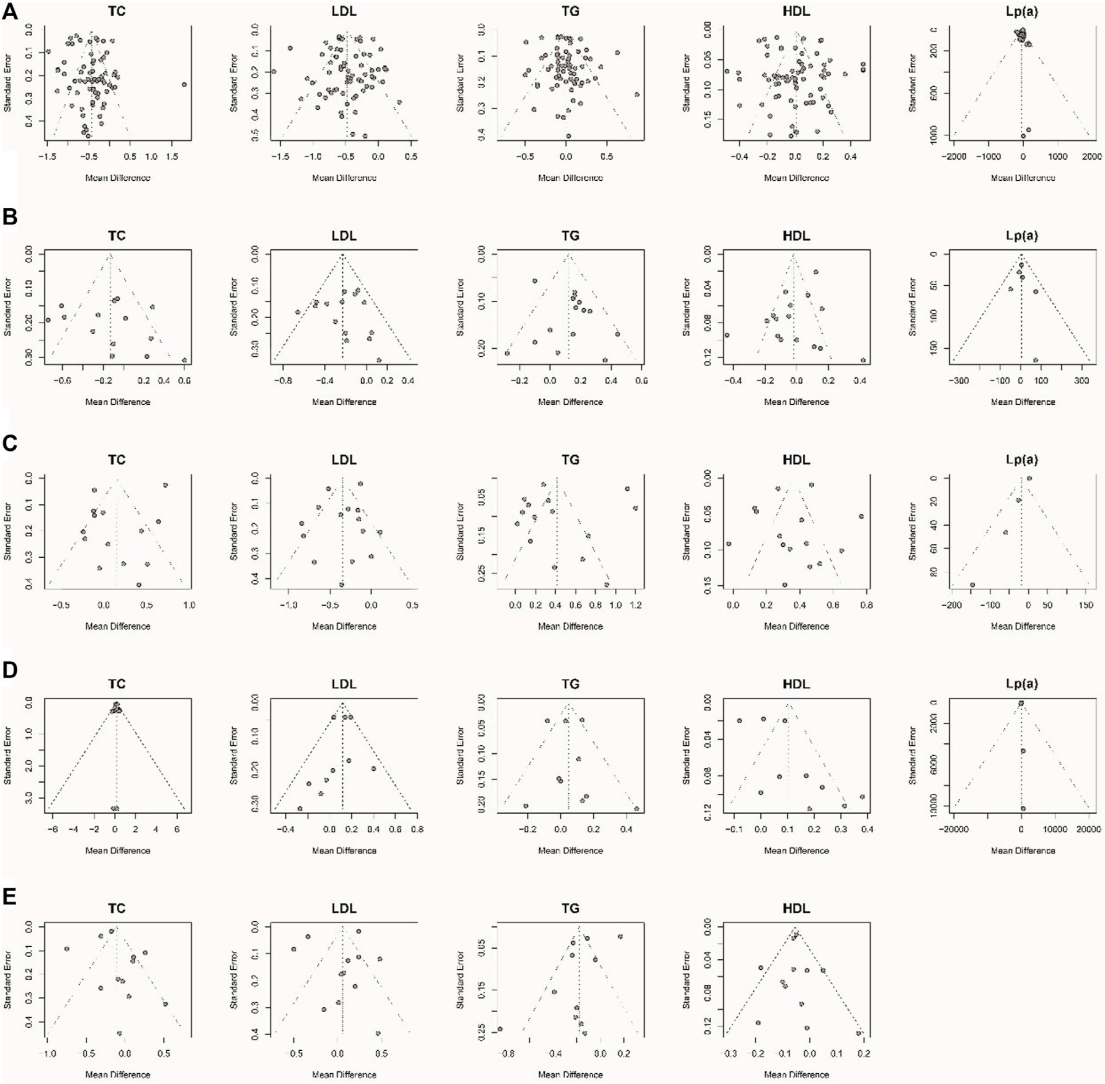
Funnel plots examining publication bias. The Egger test suggested that there was no evidence of publication bias in studies with more than 10 articles. **(A)** MHT vs. Placebo or no treatment; **(B)** oral MHT vs. transdermal MHT; **(C)** Conventional MHT vs. Tibolone; **(D)** Estrogen vs. Estrogen-Progestogen; **(E)** Low-dose MHT vs. Conventional MHT.

Considering that most of the pooled outcomes had an I^2^ greater than 50%, one-by-one exclusion was performed as a sensitivity analysis to confirm the robustness of the outcomes. While omitting the study de Kraker 2004 (de Kraker et al., 2004), low-dose estrogen seems to decrease TC significantly (MD: −0.17,95% CI: −0.31 to −0.02) (Figure 9). The cause of unstable results may be attributed to the difference type of estrogen used in this study. Also, an unstable result was found in TG of comparing E-alone and E + P regimen. When study of writing−group 1995 (Miller et al., 1995) was excluded, E + P group could significantly higher TG (MD: 0.08, 95% CI: 0.01–0.15) (Figure 10) than Estrogen alone. The longer period of using MPA may be a source of instability. Egger test and funnel plots suggested that there was little indication of publication bias in studies with more than 10 trials (Figure 11).

## Discussion

### Endogenous Sex Hormones and CVD Risk for Women

Endogenous sex hormones are involved in the pathogenesis of cardiovascular disease (CVD) in women. Studies have shown that estradiol (E2), the major form of ovarian estrogen before menopause, plays an active role in metabolic actions (Franck et al., 2013). Higher estrone levels were related to a higher brachial flow-mediated dilation (ie, better endothelial function) (Thurston et al., 2018). After menopause there is a drastic change in the endogenous hormonal milieu, with a decrease in estradiol. And the circulating estrone (E1) levels are relatively higher than E2. E1 is produced mostly by the conversion of androgens in peripheral tissues, and could be also converted from E2 by 17 *β*⁃ Hydroxysteroid dehydrogenase, E1 secretion also decreased after menopause and was equivalent to nearly 1/3 before menopause (Qureshi et al., 2020). Studies showed that higher E1 is associated with more stable plaque (Cortés Yamnia et al., 2020) and better endothelial function (Thurston et al., 2018), lower levels of E1 have been associated with increased all-cause mortality among postmenopausal women (de Padua Mansur et al., 20122012), which proved the importance of estrogen on CVD. In addition to E1 and E2, sex hormone binding globulin (SHBG) and testosterone (T) may be associated with future risk of CVD also. One study showed that a more androgenic hormone profile (i.e., higher levels of free T and lower levels of SHBG) was associated with greater Coronary Artery Calcium (CAC) progression up to 10 years in postmenopausal women (Subramanya et al., 2019). In summary, as deficiency of endogenous estrogen after menopause and the importance of estrogen for CVD, the exogenous estrogen based MHT should be benefit for CVD and related high-risk factors in theory.

### The Effects of MHT on Lipid Profile in Postmenopausal Women

Our systematic review indicated that compared with placebo or no treatment, MHT could significantly decrease the concentrations of TC, LDL-C, and Lp (a). Lp(a) is an independent risk factor for CVD and recurrent ischemic stroke (Nordestgaard et al., 2010), the previous study showed the similar result of MHT on Lp(a) with us (van Dam-Nolen et al., 2021). As for the TG concentration, previous study had showed that MHT could significantly increase it (Stevenson et al., 2015). However, no significant difference in TG between two groups was found in our study. Hence, generally speaking, MHT was associated with favorable changes in lipid parameters whether short-term or long-term using in postmenopausal women.

The bioavailability of oral estrogen is mainly low due to first-pass metabolism, which may result in adverse reactions that influence the risk of CVD. Transdermal MHT is more appropriate for cases with a high-risk of CVD or dyslipidemia than oral agents. The results of our study showed that oral MHT significantly increased TG concentration compared with transdermal MHT. In addition, a meta-analysis conducted in 2006 revealed that oral MHT adversely affected C-reactive protein (CRP) level (Ambikairajah et al., 2019). Therefore, for women with hypertriglyceridemia or other high-risk factors of CVD, transdermal route is recommended. However, oral MHT is associated with positive effects in LDL-C concentration in our study. As we know, the LDL-C concentration is the main risk factor for the occurrence and development of atherosclerosis, and was regarded as an important index to assess the risk of atherosclerotic CVD (ASCVD) (Stone et al., 2013; Jacobson et al., 2015). Hence, for women without any risk of CVD or hypertriglyceridemia, oral MHT could possibly provide greater benefits.

Considering the safety factor, the minimum effective dose of estrogen was recommanded (Menopause Subgroup, Chinese Society of Obstetrics and Gynecology, Chinese Medical Association, 2018). However, whether the low-dose MHT could achieve the same effects on lipid profile as conventional-dose MHT is still confused. One study indicated that low-dose MHT was associated with higher levels of TC and LDL-C, lower TG level (Casanova et al., 2015). Our study showed the similar benefit on TG in low-dose MHT group, but no significant difference in TC and LDL-C levels between two groups. Furthermore, low-dose MHT was found could decrease HDL-C level. Epidemiologically, a low plasma level of HDL-C was associated with an increased risk of ischemic CVD (Haase et al., 2012). Taken together, the advantage of low-dose MHT on lipid profile was possibly only confined to the TG level.

Tibolone is a synthetic hormone with estrogenic, progestogenic, and androgenic properties, and was widely used for alleviating menopausal symptoms in postmenopausal women. Tibolone has shown promising effects on improving depression and libido, and does not increase breast density (Cummings et al., 2008). As for its effects on lipid profile, a meta-analysis10 conducted in 2017 concluded that there was no significant difference between conventional MHT and Tibolone in Lp(a) concentration, which is similar to our findings. While conventional MHT was found with lower LDL-C level and higher HDL-C level compared with Tibolone, while higher TG concentration. It is suggested that tibolone is more beneficial on TG concentration.

Progestogens are indicated as a part of systemic hormone therapy in women with an intact uterus, preventing estrogen-induced endometrial hyperplasia and cancer during estrogen exposure. However, an increased risk of CHD in women receiving estrogen plus progestogen therapy rather than in those receiving CEE alone was reported (Falkeborn et al., 1992). Thus, it should be indicated whether progestogen contributes to adverse outcomes of CVD. However, no large-scale RCT has evaluated the lipid profile according to the type of progestogen used. A previous observational study revealed that the addition of progestogens blunts the lipid-related effects (Shufelt and Manson, 2021), and a meta-analysis performed in 2017 indicated that there was no significant difference in the reduction of Lp(a) concentration by E-alone compared with E + P (Anagnostis et al., 2017). The results in our study showed that E + P regimen weakened the benefits of estrogen mono-therapy. However, it should be noted that the progestogens included in our analysis were mainly composed of synthetic progestogen, and further research is required to explore whether natural progesterone could positively influence lipid profile.

Except for routine MHT, selective estrogen receptor modulators (SERMs), such as tamoxifen and raloxifene, are widely used for patients with breast cancer or osteoporosis. SERMs mimic estrogen action in certain tissues while opposing it in others. The effect of SERMs on lipids profile is also an issue worthy of attention. The meta-analysis had showed that tamoxifen can alter the lipid profile in females, particularly by decreasing TC, LDL-C and HDL–C (Alomar et al., 2022). Rraloxifene can increase HDL-C and lower LDL-C and TC (Yang et al., 2021). Thus, SERMs is beneficial to blood lipids in general.

In addition, although the result showed the positive effects of MHT on lipid profile, it needs to be emphasized that MHT is not recommended as first-line therapy for dyslipidemia or for reducing the risk of cardiovascular disease (Panagiotis et al., 2020). For postmenopausal women with carotid atherosclerosis, the prospective study had showed that total estradiol was associated with presence of vulnerable carotid plaque as well as increased risk of stroke (Glisic et al., 2018). Therefore, it is recommended to start MHT in women <60 years of age or <10 years since menopause for the beneficial effects on CVD outcomes (2019 Surveillance of Menopause, 2019; El Khoudary et al., 2020).

For dyslipidemia, the most commonly used medication is HMG-CoA reductase inhibitors (ie, statins). Statin therapy can also have effects on gonada steroidogenesis, since this process requires cholesterol as a biochemical substrate. LDL-C has been shown to be a preferential precursor for the production of ovarian steroid hormones (Grummer and Carroll, 1988). However, no reduction in E2 or E1 in postmenopausal women taking statins, despite a significant decrease in their LDL-C levels (Bairey Merz et al., 2002). But there are many studies showing an association between statin treatment and a reduction in testosterone levels (Stamerra et al., 2021). For polycystic ovary syndrome (PCOS) women, statins could decrease testosterone and Luteinizing hormone (LH)/Follicle stimulating hormone (FSH) ratio (Seyam et al., 2017), which is beneficial in treatment of PCOS. However, the role of statins for primary prevention in postmenopausal women is debated (Cangemi et al., 2017). Evidence-based data of statins for the reduction of CVD events and all-cause mortality in primary prevention in postmenopausal women is needed (El Khoudary et al., 2020).

### Limitations

The limitations of the present study should be pointed out. Firstly, among the eligible studies, few studies were specifically designed to evaluate the effects of MHT on lipid profile as the primary outcome, restricting the generalization of our findings. Secondly, the lipid profile at baseline in the majority of the included studies was almost normal, while it remained elusive whether MHT would have the similar effects on lipid profile in women with dyslipidemia. Thirdly, owing to the small sample size, the comparison between the effects of different types of progestogen on lipid profile was not comprehensively performed. Therefore, further research needs to be conducted to eliminate the above-mentioned limitations and to confirm our findings.

## Conclusion

This meta-analysis indicated that MHT plays a positive role in lipid profile in postmenopausal women. Oral MHT was more effective in reducing LDL-C level than transdermal MHT, while it increased TG concentration. E + P regimen might blunt the benefit of estrogen on lipid profile.

## Data Availability

The raw data supporting the conclusions of this article will be made available by the authors, without undue reservation.
